# Fostering successful and sustainable collaborations to advance implementation science: the adolescent HIV prevention and treatment implementation science alliance

**DOI:** 10.1002/jia2.25572

**Published:** 2020-08-31

**Authors:** Rachel Sturke, Susan Vorkoper, Linda‐Gail Bekker, Wole Ameyan, Chewe Luo, Susannah Allison, Damilola Walker, Bill Kapogiannis, Laura Guay

**Affiliations:** ^1^ Fogarty International Center National Institutes of Health Bethesda MD USA; ^2^ Desmund Tutu HIV Centre at the University of Cape Town Cape Town South Africa; ^3^ Global HIV, Hepatitis and Sexually Transmitted Infections Programmes World Health Organization Geneva Switzerland; ^4^ HIV Section Programme Division United Nations Children's Fund (UNICEF) New York NY USA; ^5^ National Institute of Mental Health National Institutes of Health Bethesda MD USA; ^6^ Eunice Kennedy Shriver National Institute of Child Health and Human Development National Institutes of Health Bethesda MD USA; ^7^ Elizabeth Glaser Pediatric AIDS Foundation Washington DC USA

**Keywords:** implementation science, adolescent, HIV, alliance, Africa

## Abstract

**Introduction:**

HIV continues to devastate the adolescent population in sub‐Saharan Africa (SSA). The complex array of interpersonal, social, structural and system‐level obstacles specific to adolescents have slowed progress in prevention and treatment of HIV in this population. The field of implementation science holds promise for addressing these challenges.

**Discussion:**

There is growing consensus that enhanced interactions between researchers and users of scientific evidence are important and necessary to tackle enduring barriers to implementation. In 2017, the Fogarty International Center launched the Adolescent HIV Prevention and Treatment Implementation Science Alliance (AHISA) to promote communication and catalyse collaboration among implementation scientists and implementers to enhance the cross‐fertilization of insights as research advances and the implementation environment evolves. This network has identified key implementation science questions for adolescent HIV, assessed how members’ research is addressing them, and is currently conducting a concept mapping exercise to more systematically identify implementation research priorities. In addition, AHSA pinpointed common challenges to addressing these questions and discussed their collective capacity to conduct implementation science using the shared learning approach of the network. Specifically, AHISA addresses challenges related to capacity building, developing mentorship, engaging stakeholders, and involving adolescents through support for training efforts and funding region‐/country‐specific networks that respond to local issues and increase implementation science capacity across SSA.

**Conclusions:**

Innovative platforms, like AHISA, that foster collaborations between implementation science researchers, policymakers and community participants to prioritizes research needs and identify and address implementation challenges can speed the translation of effective HIV interventions to benefit adolescent health.

## 1 INTRODUCTION

While HIV continues to be one of the leading causes of death for adolescents in sub‐Saharan Africa (SSA) [1], interventions to address HIV prevention and treatment in this population have had limited effectiveness especially in low‐resource settings like SSA [2]. In these settings, the benefits of proven interventions have not been fully realized because of enduring barriers to uptake, replication and scale‐up including a complex array of interpersonal, social, structural and systems‐level obstacles that often ignore developmental stages of and the many needs particular to adolescents [3].

The field of implementation science holds promise for addressing these challenges. However, critical research capacity in implementation science and deliberate and strategic efforts to facilitate collaboration, communication and relationship building among researchers and the users of research evidence are needed to make substantial advancements. Given this critical link in the context of implementation science and representing a collective commitment to the goal of reducing adolescent HIV infections, the Center for Global Health Studies at the United States (US) National Institutes of Health’s (NIH) Fogarty International Center launched the Adolescent HIV Prevention and Treatment Implementation Science Alliance (AHISA). This innovative platform was established to identify gaps and provide opportunities for enhanced communication and collaboration between implementation scientists and programme implementers and policymakers to facilitate better utilization of scientific evidence in adolescent HIV programming while simultaneously helping to ensure that research is country driven and responsive to the adolescent context. Through this approach AHISA thereby promotes scalable, empowering, and sustainable interventions. This commentary identifies both key implementation science questions and common implementation challenges related to adolescent HIV prevention and treatment research and outlines how AHISA is collectively addressing them.

## 2 DISCUSSION: ADOLESCENT HIV IMPLEMENTATION SCIENCE ALLIANCE

Eliminating HIV as a public health threat among adolescents depends heavily on successful implementation of efficacious interventions that address the context in which adolescents live. Across the HIV continuum of care, adolescents are less likely to be tested [4] and linked to care [5], have higher loss to follow‐up [6], and worse adherence [7] and viral suppression rates [8] than their adult counterparts despite evidence for appropriate treatment and care [9]. A number of factors, including youth‐friendly health services, gender‐based violence, harmful cultural and social norms, limit the uptake of health services within this population. Furthermore, many of the effective adolescent service delivery approaches and models have not been taken to scale and implemented widely.

Implementation science offers a scientific strategy that takes a holistic approach to address these barriers and is defined as the study of methods to promote the integration of research findings and evidence into health care policy and practice to achieve their potential public health impact [10]. The intent of implementation science is to investigate and address major social, behavioural, economic and management bottlenecks that impede effective implementation, test novel approaches to improve health programming and determine the effect of implementation strategies on the causal relationship between the intervention and its impact in order to enable proven intervention to be taken to scale.

Premised on the idea that support for implementation science alone will have limited impact unless coupled with concerted efforts to bring researchers together with policymakers and programme implementers, AHISA teams are made up of researchers with funding from across the NIH and their in‐country, implementing partners. AHISA, which is funded by the US Office of the Global AIDS Coordinator (OGAC) and the NIH’s Office of AIDS Research, was developed as an innovative platform to enhance communication and catalyse collaboration among the funded implementation science researchers and users of research evidence to promote a cross‐fertilization of ideas, insights and experiences in real time as the research progresses and the implementation environment evolves. This innovative platform also helps to seed significant and sustainable implementation science collaborations aimed at addressing critical barriers to implementing proven health interventions among adolescents. Because collaboration between researchers and those who utilize research evidence is an implicit necessity for the field of implementation science, this opportunity to develop formal and informal networks is of particular importance to moving the implementation science agenda for adolescent HIV forward. Building multidisciplinary and multi‐sector implementation science collaborations will inform global initiatives (e.g. PEPFAR DREAMS, the Global Fund’s Strategic Investment in Adolescent Girls and Young Women, World Health Organization’s Global Accelerated Action for the Health of Adolescents and UNAIDS Start Free, Stay Free, AIDS Free) and help identify and overcome the intractable barriers that drive high rates of HIV among adolescents in SSA.

AHISA is made up of 26 teams of NIH‐funded researchers, programme implementers and policymakers working in 11 countries in SSA and includes over 75 members [11]. The alliance is guided by a steering committee of experts from the NIH, OGAC, US Agency for International Development, the US Centers for Disease Control and Prevention, the Elizabeth Glaser Pediatric AIDS Foundation, World Health Organization, UNICEF, and the Desmond Tutu Foundation. In addition to annual in‐person meetings, webinars and online discussion, AHISA has also funded several small collaborative awards with the goal of catalysing long‐term and sustainable region‐/country‐specific collaborations that respond to local issues and increase implementation science capacity across SSA. These small investments allow AHISA members to leverage their current research and engage a local audience that the larger alliance may not otherwise reach.

Pulling from the collective experience of its members, AHISA has identified critical implementation research questions that are imperative to moving the field of adolescent HIV prevention and treatment forward (Table 1). While researchers across the field including AHISA teams are addressing these priority areas such as transitions to adult care, predictors to uptake of and adherence to pre‐exposure prophylaxis (PrEP), integrating mental health services in clinical care of adolescents with HIV, additional implementation research is needed for effective prevention, screening and treatment of HIV among adolescents [12, 13, 14, 15, 16, 17, 18, 19, 20, 21, 22, 23]. Based on priority research questions identified by AHISA, the Eunice Kennedy Shriver National Institute of Child Health and Human Development established a consortium entitled the “Prevention and Treatment through a Comprehensive Care Continuum for HIV‐affected Adolescents in Resource Constrained Settings (PATC^3^H),” which funds eight research teams to conduct clinical research and evaluation of a variety of combination interventions aimed at the individual, family, community, structural and education, and health systems levels, to improve health outcomes among adolescents at risk for or living with HIV [24].

**Table 1:** Priority research areas for adolescent HIV

Transitions to adult care Predictors of uptake of and adherence to PrEP Integrating mental health services in clinical care of adolescents with HIV Implementation/scale up/roll out/experiences with novel treatment options Scale‐up and disseminating of pilot results (e.g. pilot test for young men who have sex with men in Nigeria) Drawing lessons from implementation science research to reach the most vulnerable and improve prevention outcomes Acceptability and adaptation of mHealth approaches to prevention and treatment of HIV among adolescents Understanding cultural barriers to the use of HIV Prevention methods among adolescent girls and young women and their partners Optimizing youth engagement Participation to improve HIV care continuum outcomes among young people. Expanding access and reach of youth‐friendly sexual and reproductive health services Engaging men and boys Increasing uptake and retention into care using differentiated service delivery model

PrEP, pre‐exposure prophylaxis.

To more systematically identify implementation research priorities for adolescent HIV, AHISA is harnessing the collective expertise and experience of its members and conducting a concept mapping exercise [25]. Specifically, this exercise uses a rigorous methodology to identify factors that impact the implementation of HIV prevention and intervention programmes for adolescents in SSA. Using a multi‐step approach, AHISA members generated responses to the focus question: “In your experience, what factors have facilitated or hindered implementation of evidence‐based HIV prevention or treatment for adolescents in SSA?” These statements were sorted into thematically relevant groups and rated each statement on importance and changeability. The exercise will result in a set of agreed upon key factors that facilitate or hinder implementation of evidence‐based HIV prevention or treatment for adolescents in SSA; factors that could be addressed by implementation science.

In addition to research priorities, AHISA members have identified common challenges to addressing these questions and discussed their collective capacity to conduct implementation science using the shared learning approach of the network. AHISA has outlined and addressed a range of challenges related to implementation science and adolescent HIV research:

### 2.1 Building capacity in implementation science

Lack of capacity to conduct implementation research exists in the region and global health more generally. To help build implementation science capacity, AHISA in collaboration with the University of North Carolina and Wits University hosted a three‐day training to provide alliance members with an in‐depth overview of the theory, operational and evaluation approaches to implementation science; methods to assess barriers in implementation science and strategies/tools to overcome them; and options for dissemination of results [26, 27].

To further develop tools for sustainable implementation science capacity, through the collaborative awards, AHISA is supporting the development of the Adolescents in Research Toolkit which will source and consolidate information pertaining to clinical, research and ethico‐legal aspects of HIV prevention and treatment clinical trials in low‐ and middle‐income countries and share this information through an online toolkit aimed at a wide variety of stakeholders working within adolescent health research and who are designing, implementing and disseminating adolescent health research and implementation science projects.

AHISA continues to build the capacity through trainings at each of the annual meetings and through biannual webinars on topics related to implementation science and adolescent HIV with the aim of strengthening learning among members. These activities allow members to both increase their capacity for conducting implementation science and share relevant work in the field demonstrating the applicability of implementation science within their own research.

### 2.2 Developing mentorship

Given the lack of capacity in implementation science, there are insufficient mentors for the discipline especially in SSA. These mentors are needed to build a sustainable pipeline of independent implementation scientists. To help address this challenge, AHISA has developed opportunities for mentoring through ongoing engagement with implementation science experts and continued capacity building. For example the AHISA‐supported Central and West Africa Implementation Science Alliance (CAWISA), a locally led alliance with membership from Ghana, Nigeria, Cameroon and the Democratic Republic of the Congo, is supporting mentoring relationships by pairing early investigator mentees with both a senior research member of the CAWISA steering committee and a representative from a local institution for between two and five years. In addition, CAWISA aims to develop a mentorship toolkit as the programme progresses.

### 2.3 Engaging stakeholders

There are important challenges in engaging stakeholders throughout the research process. First, researchers and policymakers ask different questions. Most researchers ask questions rooted in a quest for empirical evidence, whereas policymakers are often looking for programmatic/public health insights to inform policy and programme changes. Second, researchers and decision makers have different stakeholder audiences. While researchers often pursue broad scientific theory and work with a view towards generalizable findings, policymakers need answers to questions that are targeted to their distinct population. Finally, there is an important tension between the long gestation periods and rigorous requirements of research and the short time frames and need for quick information for policymakers and implementers. Strategies to help reconcile these time frames are critical. Recognizing the critical differences between the issues researchers and decision makers prioritize, AHISA has explored strategies for stakeholder engagement and has hosted several discussions with policymaking and programme implementing partners to understand best practices. Overall, successful research and practice partnerships emphasized the need to engage various stakeholders early in the research process to better align each other’s interests and motivation and to include these stakeholders in the process to ensure timely dissemination of the results.

In addition, four collaborative AHISA‐sponsored small awards support country‐specific alliances – in Kenya, South Africa, Uganda and Zambia – that develop collaborative partnerships with other AHISA teams also engage local stakeholders, including policymakers, programme implementers and youth. By encouraging a more local geographic focus, these alliances are able to address specific local policies, such as Kenya’s *Fast‐Track Plan to End HIV and AIDS among Adolescents and Young People*, tackle areas of particular concern for a country, such as Uganda’s HIV service delivery for adolescents, and focus on more granular populations.

### 2.4 Involving adolescents

Adolescents themselves are important stakeholders often left out of the research process. They are critical to understanding barriers to implementation as well as to designing effective implementation science studies that address challenges in the context of this unique population. Without their engagement, uptake and feasibility of interventions is severely compromised. Indeed, adolescents can play important roles as advocates, participants and advisors to research. To increase the input of adolescents, AHISA has sponsored youth participants at the 2018 International AIDS Society conference, which gathered over 20,000 participants from more than 170 countries. In addition, AHISA supports the African Youth Implementation Science Alliance, which is developing a cadre of young researchers under 30 years old to build their capacity in implementation science. Along with engaging youth, a key component of the Youth Alliance is to provide both training and ongoing mentorship to ensure that young researchers are supported in the appropriate use of the implementation science designs and frameworks thereby ensuring that their results are efficacious and replicable.

While most of these challenges call for long‐term investments, AHISA enables its members to share best practices and novel approaches while supporting the development of creative, empowering, and sustainable collaborations and implementation science capacity.

## 3 CONCLUSIONS

The work of the alliance is well positioned to inform local and national policies and programmes related to adolescent HIV. For example research being conducted in Kenya in collaboration with the National AIDS and STI's Control Programme is developing a novel approach to address the continuum between paediatric to adult HIV care that can be scaled nationally [28]. Similarly, a partnership between researchers and the Rwanda Biomedical Center is primed to increase ART adherence among young people living with HIV [29]. Research focused on the challenges of effective and sustainable implementation of proven interventions in real‐world settings is critical to the goal of achieving an AIDS‐free generation and ensuring the survival and health of adolescents. Integration of clinical research evidence, programme implementation, and policy is critical to speed the translation of effective HIV prevention and treatment interventions. Implementation science can help close the gap between evidence and programme and policy as well as address stubborn barriers. Innovative platforms, like AHISA, that bring together and foster collaboration of implementation science researchers with implementers, policymakers and community participants prioritize research needs and identify and address implementation challenges that can speed the translation of effective HIV interventions to benefit adolescent health. Indeed, AHISA helps create and enhance partnerships, galvanizes regional and country investment and ownership, builds regional research capacity in implementation science, and can help promote meaningful engagement of adolescents in helping to overcome implementation challenges.

## COMPETING INTERESTS

There are no competing interests.

## AUTHORS’ CONTRIBUTIONS

RS and SV involved in conception of the work and drafting the article. RS, SV, LGB, WA, CL, SA, DW, BK and LG carried out final approval of the version to be published.

## ABBREVIATIONS

AHISA, Adolescent HIV Prevention and Treatment Implementation Science Alliance; CAWISA, Central and West Africa Implementation Science Alliance; NIH, United States National Institutes of Health; OGAC, US Office of the Global AIDS Coordinator; PATC^3^H, Prevention and Treatment through a Comprehensive Care Continuum for HIV‐affected Adolescents in Resource Constrained Settings; PrEP, pre‐exposure prophylaxis; SSA, sub‐Saharan Africa.

## AUTHORS’ INFORMATION

All authors are part of the AHISA Steering Committee.
